# Hospital level variations in the trends and outcomes of the nonoperative management of splenic injuries – a nationwide cohort study

**DOI:** 10.1186/s13049-018-0578-y

**Published:** 2019-01-11

**Authors:** Chien-An Liao, Yu-Tung Wu, Chien-Hung Liao, Shang-Yu Wang, Chih-Yuan Fu, Chi-Hsun Hsieh, Shao-Wei Chen, Ching-Chang Chen, An-Hsun Chou, Chi-Tung Cheng

**Affiliations:** 1 Division of Trauma and Emergency Surgery, Department of Surgery, Chang Gung Memorial Hospital, Linkou, Taoyuan City, Taiwan; 2 Division of Thoracic and Cardiovascular Surgery, Department of Surgery, Chang Gung Memorial Hospital, Taoyuan City, Taiwan; 3 Department of Neurosurgery, Chang Gung Memorial Hospital, Linkou Medical Center, Chang Gung University, Taoyuan City, Taiwan; 4 Department of Anesthesiology, Chang Gung Memorial Hospital, Linkou Medical Center, Chang Gung University, 5 Fu-Hsing Street, Kwei-Shan Shiang, Taoyuan, Taiwan; 5 Department of Surgery, Chang Gung Memorial Hospital, Chang Gung University, 5 Fu-Hsing Street, Kwei-Shan Shiang, Taoyuan, Taiwan; 6 Institute of Biomedical Informatics, National Yang-Ming University, Taipei, Taiwan

**Keywords:** Non-operative treatment, Spleen injury, Hospital level

## Abstract

**Background:**

The long-term treatment trends of splenic injuries can provide guidance when treating trauma patients. The nonoperative management (NOM) of splenic injuries was introduced in early 1989. After decades of development, it has proven to be safe and is now the primary treatment choice worldwide. However, there remains a lack of nationwide registry data to support the feasibility and efficiency of NOM.

**Methods:**

We used the Taiwan National Health Insurance Research Database to conduct a whole population-based cohort study. Patients admitted with blunt splenic injuries from 2002 to 2013 were identified. Demographic data, management methods, associated injuries, comorbidities and outcome parameters were collected. Patients were divided into 2 groups by the type of admitting institution: a tertiary center or a non-center hospital. We also used 4 years as an interval to analyze the changes in epidemiological data and treatment trends. Comparisons of the results of NOM and surgical management were also performed.

**Results:**

A total of 12,455 patients were admitted with blunt splenic injuries between 2002 and 2013. Among the 11,551 patients treated in a single hospital after admission, patients underwent NOM more frequently at tertiary centers than at non-center hospitals (64.6% vs 50.3%). During the 12-year study period, the NOM rate increased from 56 to 73% in tertiary centers, while in noncenter hospitals, the rate only increased from 43 to 58%. The mortality rate decreased in tertiary centers from 8.9 to 7.2%, with no apparent change in noncenter hospitals. Complications occurred more frequently in the surgical management group.

**Conclusion:**

There is a trend toward the use of NOM for blunt splenic injury treatments, and the outcomes from the NOM groups were not inferior to those of the operation group. In addition, tertiary centers performed more NOM than did non-center hospitals and better met the international consensus.

**Electronic supplementary material:**

The online version of this article (10.1186/s13049-018-0578-y) contains supplementary material, which is available to authorized users.

## Background

The nonoperative management (NOM) of blunt splenic injuries has been established as a reliable and effective method [1]. NOM was first introduced for pediatric blunt abdominal trauma and then extended for use in adult trauma in the early 1990s. The pediatric outcomes are very satisfactory, [2] and several clinical factors such as the patient’s age, splenic injury grade, and availability of emergent radiological interventions have been discussed regarding the successful NOM rate in splenic injuries [3]. Other studies have focused on hospital factors such as patient volume, grading and insurance type.

The first large-scale study of treatment outcomes and trends for NOM was published in 1997. At that time, the NOM rate was approximately 50%, with a 13% mortality rate and a higher failure rate in geriatric patients [4]. In addition, NOM had replaced splenorrhaphy for spleen preservation management in 65% of patients with blunt trauma, with a success rate of over 90% [5]. Recent studies revealed even higher NOM rates of up to 70 to 80% in patients with blunt splenic injuries, with sustained good outcomes [6]. There was also a study that showed a NOM rate of over 90%, with success in 90% of the cases [7].

However, few studies have been published using global registry datasets, and it is unknown whether NOM is appropriate for all institutes or should be limited to specific trauma centers. Therefore, we conducted the largest study using the Taiwan National Health Insurance Research Database (NHIRD) to assess the treatment trends and clinical outcomes of splenic injury.

## Materials and methods

### Data source

Data for this study was obtained from the Taiwan NHIRD. The National Health Insurance (NHI) program in Taiwan started on March 1, 1995 and covers more than 99% of Taiwan’s population. All the registration files and original claim data for reimbursement purposes at the hospitals are recorded in the NHIRD. To protect patient privacy, the dataset was de-identified and anonymized. The database contains all the admission records, diagnosis codes, hospital information and procedures received by each patient. The NHI procedure codes are the basis of the institute’s claims for government payment. There is an independent peer review system for the identification of procedures based on medical records. Hospitals are accredited by the Joint Commission of Taiwan and re-evaluated every 4 years according to the Hospital Accreditation Scheme from the Ministry of Health and Welfare; hospitals are generally divided into three levels including tertiary centers, regional hospitals and district hospitals. An accredited hospital is downgraded if it does not meet the criteria and service quality requirements of the previous level. The level of a hospital is associated with medical service payments, and some procedures are limited in tertiary centers. This study was exempt from full review by the Ethics Institutional Review Board of Chang Gung Memorial Hospital.

### Study population

In this study, we used all the admission records between 1997 and 2013 for analysis. We identified all the patients who were admitted between January 2002 and December 2013 with a diagnosis of splenic injury (International Classification of Diseases, Ninth Revision, Clinical Modification [ICD-9CM] code 865). Patients with penetrating splenic injuries were excluded (ICD-9CM code 865.1). All the patients younger than 18 years old at the index admission were also excluded. All the NHI procedure codes used during the admission period were analyzed. Patients who underwent splenectomy, splenorrhaphy and partial splenectomy were defined as the surgical management group; the other patients were defined as the NOM group. The hospital level was divided into tertiary centers and noncenter hospitals according to the Hospital Accreditation Scheme from the Ministry of Health and Welfare. The injury severity score (ISS) and abbreviated injury scale (AIS) scores are not included in the NHIRD, and an alternative method of evaluating the severity of an illness consists of using the “catastrophic illness card” that is certified by the government. NHI program identifies patients with an ISS ≥ 16 as having a major illness, and they are provided payment relief. We used this information to analyze the illness severity, and these patients are categorized as ISS ≥ 16 in our study. In Taiwan, a trauma center system is not included in the health policy. However, there is a similar system called emergency medical ability classification that includes trauma management. A tertiary center must be qualified for the highest class of emergency medical ability to obtain this classification.

### Associated injuries, comorbidities and outcomes

Major associated injuries from the same trauma episode, including traumatic brain injury, cardiopulmonary injury, hemothorax, gastrointestinal injury, kidney injury, liver injury, pelvic fracture, femoral fracture and spinal fracture, were analyzed. The underlying comorbidities were identified according to previous admission records and the index admission of trauma including diabetes mellitus, hypertension, coronary artery disease, chronic obstructive pulmonary disease, cirrhosis, chronic kidney disease and cancer. The blood product transfusion volume, hospital length of stay, intensive care unit (ICU) length of stay, ventilator support days and in-hospital mortality of index trauma admission were defined as outcome parameters. The definition of the above diagnoses are described in Additional file 1: Table S1.

### Statistical analysis

All the analyses were performed using R (v3.4.1). Continuous variables were analyzed by the Kruskal-Wallis rank sum test, and categorical variables were analyzed by chi-square tests. Trends were analyzed with the Cochran-Armitage test. All the statistical tests were two-sided, and *p-*values < 0.05 were considered statistically significant.

## Results

The basic demographic data of patients with blunt splenic injuries during the study period in Taiwan are shown in Table 1. A total of 12,455 patients with a median age of 37.7 years were identified. Of these patients, 39.6% were treated in a tertiary center or transferred to a tertiary center after initial stabilization in the emergency department of another institute, and 7.3% were transferred from another hospital after admission. A total of 43.7% of patients received surgical management, and the overall mortality rate was 7.8%.

**Table 1:** Demographic data of patients with splenic injuries, *n* = 12,455

Gender		
Male	8805	(70.7%)
Female	3650	(29.3%)
Age (years, median [IQR])	37.74	[24.15, 52.69]
Hospital level		
Tertiary center	4926	(39.6%)
Noncenter hospital	7529	(60.4%)
Transfer after admission		
Yes	904	(7.3%)
No	11,551	(92.7%)
Management		
Non-operative	7018	(56.3%)
Surgical	5437	(43.7%)
Associated injury		
Traumatic brain injury	2220	(17.8%)
Cardiopulmonary injury	729	(5.9%)
Hemothorax	2173	(17.4%)
Gastrointestinal injury	1084	(8.7%)
Kidney injury	1356	(10.9%)
Liver injury	1596	(12.8%)
Pelvic fracture	549	(4.4%)
Femoral fracture	555	(4.5%)
Spinal fracture	487	(3.9%)
Underlying disease		
Diabetes mellitus	846	(6.8%)
Hypertension	1210	(9.7%)
Coronary artery disease	399	(3.2%)
COPD	242	(1.9%)
Cirrhosis	558	(4.5%)
Chronic kidney disease	91	(0.7%)
Cancer	249	(2.0%)
Blood transfusion (unit)^a^ (median [IQR])		
PRBC	4	[0.00, 8.00]
FFP	0	[0.00, 6.00]
Platelet	0	[0.00, 0.00]
WB	0	[0.00, 0.00]
Hospital length of stay (days) (median [IQR])	9	[6.00, 14.00]
ICU length of stay (days) (median [IQR])	2	[1.00, 4.00]
Ventilator support days (median [IQR])	0	[0.00, 2.00]
In-hospital mortality	977	(7.8%)

*IQR* interquartile range, *COPD* chronic obstructive pulmonary disease, *PRBC* packed red blood cell, *FFP* fresh frozen plasma, *WB* whole blood

^a^Taiwan unit, blood product separated from 250 ml of whole blood

**Fig. 1:**
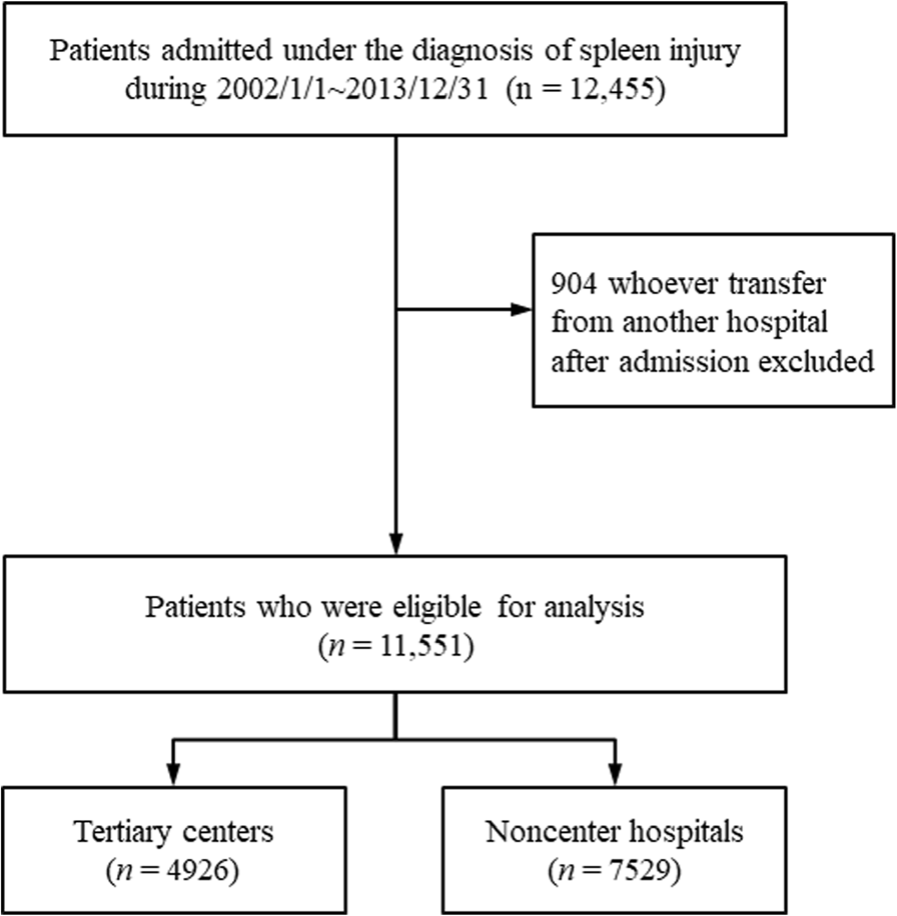
The flow chart of patient enrollment and study design

We excluded patients who had transferred from another hospital after admission to eliminate differences between hospitals, leaving a total of 11,551 patients who were treated in a single hospital after admission. These patients were divided into 2 groups by the hospital where they received definite treatment: tertiary centers and noncenter hospitals. A total of 39.3% of the patients were treated in tertiary centers (Fig. 1).

The NOM rate at the tertiary centers was significantly higher than that in noncenter hospitals (64.6% vs 50.3%). On the other hand, the incidence of associated injuries, including cardiopulmonary injury (7.7% vs 4.1%), hemothorax (18.7% vs 15.1%), kidney injury (12.2% vs 9.9%), pelvic fracture (4.6% vs 3.4%), spinal fracture (4.5% vs 2.7%) and femoral fracture (4.8% vs 3.7%), was significantly higher in the tertiary center group.

Because the patient characteristics and medical resources differed at the tertiary centers and noncenter hospitals, we analyzed the demographic data trends by hospital level. We separated the 12-year period into 3 equal periods to compare demographic factors, treatments and outcomes between each period; these data are shown in Table 2. During the study period, the surgical treatment rate for blunt splenic injuries dropped significantly from 43.6 to 27.4% in tertiary centers. The associated gastrointestinal and cardiopulmonary injuries increased, and spinal fractures decreased, while other injuries remained similar. Although the NOM rate increased, the overall mortality rate dropped from 8.9 to 7.2%. The hospital length of stay gradually decreased, and the ICU length of stay slightly increased.

**Table 2:** Comparison of the characteristics of splenic injury patients by hospital level and year of injury (*n* = 11,551)

	2002–2005		2006–2009		2010–2013			2002–2005		2006–2009		2010–2013		
	Tertiary Center						*p* value	Non-center						*p* value
Gender							0.106							0.081
Male	1057	(73.0%)	1070	(70.4%)	1056	(69.6%)		1631	(71.2%)	1696	(71.5%)	1652	(68.8%)	
Female	391	(27.0%)	450	(29.6%)	461	(30.4%)		660	(28.8%)	677	(28.5%)	750	(31.2%)	
Age group							0.262							0.079
18–40	862	(59.5%)	850	(55.9%)	854	(56.3%)		1236	(54.0%)	1205	(50.8%)	1203	(50.1%)	
40–65	452	(31.2%)	517	(34.0%)	521	(34.3%)		797	(34.8%)	868	(36.6%)	896	(37.3%)	
> 65	134	(9.3%)	153	(10.1%)	142	(9.4%)		258	(11.3%)	300	(12.6%)	303	(12.6%)	
Management							< 0.001^*^							< 0.001^*^
Non-operative	816	(56.4%)	987	(64.9%)	1101	(72.6%)		994	(43.4%)	1207	(50.9%)	1392	(58.0%)	
Surgical	632	(43.6%)	533	(35.1%)	416	(27.4%)		1297	(56.6%)	1166	(49.1%)	1010	(42.0%)	
Associated injury														
Traumatic brain injury	248	(17.1%)	279	(18.4%)	248	(16.3%)	0.337	421	(18.4%)	360	(15.2%)	388	(16.2%)	0.011^*^
Cardiopulmonary injury	63	(4.4%)	118	(7.8%)	165	(10.9%)	< 0.001^*^	57	(2.5%)	106	(4.5%)	130	(5.4%)	< 0.001^*^
Hemothorax	245	(16.9%)	288	(18.9%)	305	(20.1%)	0.08	260	(11.3%)	377	(15.9%)	433	(18.0%)	< 0.001^*^
Gastrointestinal injury	147	(10.2%)	122	(8.0%)	91	(6.0%)	< 0.001^*^	238	(10.4%)	183	(7.7%)	179	(7.5%)	< 0.001^*^
Kidney injury	168	(11.6%)	192	(12.6%)	186	(12.3%)	0.687	225	(9.8%)	222	(9.4%)	252	(10.5%)	0.417
Liver injury	179	(12.4%)	211	(13.9%)	170	(11.2%)	0.082	304	(13.3%)	288	(12.1%)	268	(11.2%)	0.086
Pelvic fracture	70	(4.8%)	76	(5.0%)	63	(4.2%)	0.503	71	(3.1%)	87	(3.7%)	84	(3.5%)	0.551
Femoral fracture	80	(5.5%)	71	(4.7%)	68	(4.5%)	0.376	79	(3.4%)	82	(3.5%)	103	(4.3%)	0.214
Spinal fracture	51	(3.5%)	65	(4.3%)	86	(5.7%)	0.016^*^	46	(2.0%)	57	(2.4%)	89	(3.7%)	0.001^*^
Hospital length of stay days (median [IQR])	10	[6, 15]	9	[6, 16]	9	[6, 15]	0.005^*^	9	[6, 14]	9	[6, 14]	9	[6, 13]	0.116
ICU length of stay days (median [IQR])	2	[1, 4]	2	[1, 4]	2	[1, 4]	0.029^*^	2	[0, 4]	2	[1, 4]	2	[1, 4]	0.001^*^
Ventilator support days(median [IQR])	0	[0, 2]	0	[0, 2]	0	[0, 2]	0.002^*^	0	[0, 1]	0	[0, 2]	0	[0, 2]	0.03^*^
In-hospital mortality	129	(8.9%)	143	(9.4%)	109	(7.2%)	0.071	172	(7.5%)	208	(8.8%)	184	(7.7%)	0.221
ISS ≥ 16	192	(13.3%)	398	(26.2%)	547	(36.1%)	< 0.001^*^	224	(9.8%)	328	(13.8%)	520	(21.6%)	< 0.001^*^

*COPD* chronic obstructive pulmonary disease, *IQR* interquartile range

^*^Statistically significant difference

The trends for treatments in the noncenter hospital group showed similar patterns of increasing NOM, but the surgical management rate remained as high as 42.0% over the last 4 years. The demographic distribution trends for age, gender, and associated injuries were similar to those of the tertiary center group. However, there was no significant change in the overall mortality rate. The hospital length of stay and ICU length of stay remained similar and did not show similar patterns of change to those seen in the tertiary center group.

**Fig. 2:**
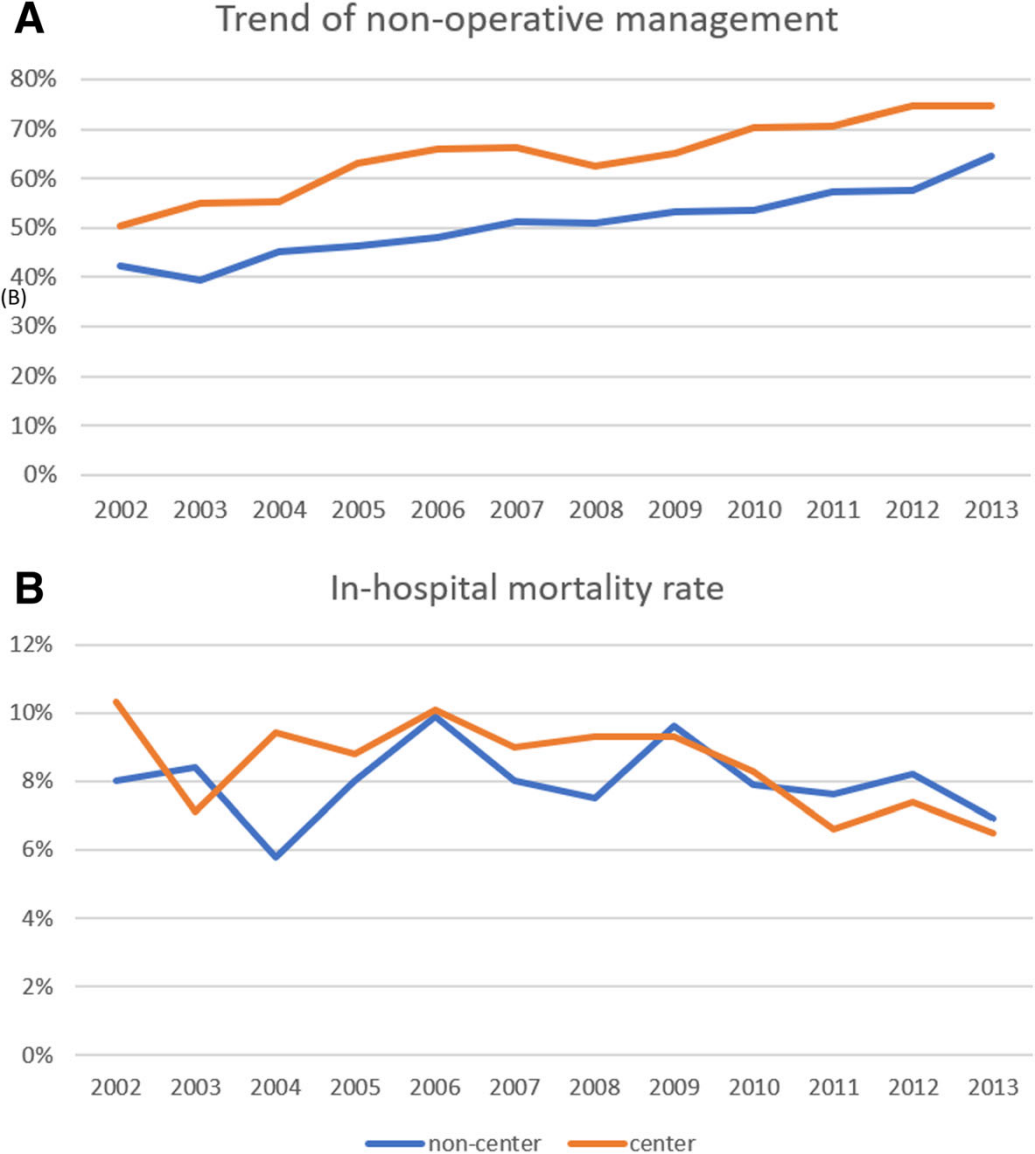
(**a**) The trend of non-operative management from 2002 to 2013 divided by tertiary center and non-center group. (**b**) The trend of in-hospital mortality rate during 2002 to 2013 divided by tertiary center and non-center group

The trends for the NOM and mortality rates are shown in Fig. 2a and b, respectively. The NOM rate gradually decreased in both tertiary centers and noncenter hospitals (trend test, *p* < 0.001). The mortality rate in the tertiary centers decreased slightly during the study period, but the trend test was not significant (*p* = 0.1267); there were no significant changes in the non-center group (*p* = 0.9131).

We compared the NOM and surgical management groups in Table 3. The nonoperative group was younger, and NOM was performed more frequently in tertiary centers. Regarding the associated injuries, traumatic brain injury, gastrointestinal tract injury, liver injury and femoral fracture were significantly more frequent in the surgical management group, while cardiopulmonary injury, kidney injury, pelvic fracture and spinal fracture were more frequent in the NOM group. Clinical outcomes including blood transfusion volume, in-hospital mortality rate, lengths of stay in the hospital and ICU and ventilator support days were worse in the operative group. Complications such as gastrointestinal bleeding, wound infection and secondary pneumonia were also higher in the surgical management group. The percentage of patients with an ISS ≥ 16 was also higher in the surgery group than in the NOM group (24.8% vs 14.7%, *p* < 0.001). These data support current concept that severity is positively correlated with surgical intervention.

**Table 3:** Comparison of the characteristics of splenic injury patients by management

	Non-operative management *n* = 6497		Surgical management *n* = 5054		*p* value
Gender					0.031^*^
Male	4538	(69.8%)	3624	(71.7%)	
Female	1959	(30.2%)	1430	(28.3%)	
Age group					0.003^*^
18–40	3538	(54.5%)	2672	(52.9%)	
40–65	2290	(35.2%)	1761	(34.8%)	
> 65	669	(10.3%)	621	(12.3%)	
Hospital level					< 0.001^*^
Tertiary center	2904	(44.7%)	1581	(31.3%)	
Non-center	3593	(55.3%)	3473	(68.7%)	
Associated injury					
Traumatic brain injury	1029	(15.8%)	915	(18.1%)	0.001^*^
Cardiopulmonary injury	388	(6.0%)	251	(5.0%)	0.021^*^
Hemothorax	1091	(16.8%)	817	(16.2%)	0.382
Gastrointestinal injury	265	(4.1%)	695	(13.8%)	< 0.001^*^
Kidney injury	812	(12.5%)	433	(8.6%)	< 0.001^*^
Liver injury	729	(11.2%)	691	(13.7%)	< 0.001^*^
Pelvic fracture	279	(4.3%)	172	(3.4%)	0.016^*^
Femoral fracture	227	(3.5%)	256	(5.1%)	< 0.001^*^
Spinal fracture	281	(4.3%)	113	(2.2%)	< 0.001^*^
Total blood transfusion (unit)^a^ median [IQR]					
PRBC	0	[0, 4]	8	[4, 14]	< 0.001^*^
FFP	0	[0, 0]	4	[0, 12]	< 0.001^*^
Platelet	0	[0, 0]	0	[0, 0]	< 0.001^*^
WB	0	[0,0]	0	[0, 0]	< 0.001^*^
Hospital stay (days) median [IQR]	8	[5, 12]	11	[8, 17]	< 0.001^*^
ICU stay (days) median [IQR]	2	[0, 3]	3	[1, 5]	< 0.001^*^
Ventilator support days median [IQR]	0	[0, 0]	1	[0, 4]	< 0.001^*^
Complications					
Pneumonia	94	(1.4%)	103	(2.0%)	0.018^*^
Sepsis	150	(2.3%)	109	(2.2%)	0.628
Wound infection	23	(0.4%)	33	(0.7%)	0.031^*^
Stroke	13	(0.2%)	19	(0.4%)	0.108
Gastrointestinal bleeding	119	(1.8%)	133	(2.6%)	0.004^*^
In-hospital mortality	350	(5.4%)	595	(11.8%)	< 0.001^*^
ISS > 16	954	(14.7%)	1255	(24.8%)	< 0.001^*^

*PRBC* packed red blood cell, *FFP* fresh frozen plasma, *WB* whole blood, *IQR* interquartile range

^*^Statistically significant difference

^a^Taiwan unit, blood product separated from 250 ml of whole blood

We then conducted a multivariate analysis of mortality rates. Table 4 shows that the only factors associated with mortality are patient age, associated injuries such as traumatic brain injury or liver injury and underlying clinical conditions such as liver cirrhosis and hemodialysis. The hospital level and use of NOM did not influence the clinical outcomes.

**Table 4:** Risk factor analysis of in-hospital mortality of patients with splenic injuries

	Univariate analysis					Multivariate analysis	
	Non-operative management		operative management		*p* value	Odds ratio	*p* value
Gender					0.867		
Male	7497	(70.7%)	665	(70.4%)		1	
Female	3109	(29.3%)	280	(29.6%)		1.004 (0.842–1.195)	0.961
Age group					< 0.001^*^		
18–40	5851	(55.2%)	359	(38.0%)		1	
40–65	3706	(34.9%)	345	(36.5%)		1.572 (1.302–1.897)	**< 0.001** ^*^
> 65	1049	(9.9%)	241	(25.5%)		4.228 (3.336–5.356)	**< 0.001** ^*^
Hospital level					0.344		
Tertiary center	4104	(38.7%)	381	(40.3%)		1	
Non-center hospital	6502	(61.3%)	564	(59.7%)		0.943 (0.801–1.111)	0.484
Management					< 0.001^*^		
Non-operative	6147	(58.0%)	350	(37.0%)		1	
Surgical	4459	(42.0%)	595	(63.0%)		0.974 (0.813–1.166)	0.777
Associated injury							
Traumatic brain injury	1546	(14.6%)	398	(42.1%)	< 0.001^*^	4.693 (3.963–5.558)	**< 0.001** ^*^
Cardiopulmonary injury	546	(5.1%)	93	(9.8%)	< 0.001^*^	2.078 (1.566–2.734)	**< 0.001** ^*^
Hemothorax	1667	(15.7%)	241	(25.5%)	< 0.001^*^	1.474 (1.219–1.777)	**< 0.001** ^*^
Gastrointestinal injury	820	(7.7%)	140	(14.8%)	< 0.001^*^	1.579 (1.240–2.001)	**< 0.001** ^*^
Kidney injury	1141	(10.8%)	104	(11.0%)	0.857	1.237 (0.958–1.584)	0.097
Liver injury	1157	(10.9%)	263	(27.8%)	< 0.001^*^	2.694 (2.224–3.259)	**< 0.001** ^*^
Pelvic fracture	378	(3.6%)	73	(7.7%)	< 0.001^*^	1.941 (1.406–2.652)	**< 0.001** ^*^
Femoral fracture	420	(4.0%)	63	(6.7%)	< 0.001^*^	1.028 (0.731–1.428)	0.870
Spine fracture	357	(3.4%)	37	(3.9%)	0.425	0.857 (0.559–1.282)	0.465
Underlying disease							
Diabetes mellitus	655	(6.2%)	97	(10.3%)	< 0.001^*^	0.955 (0.706–1.281)	0.761
Hypertension	944	(8.9%)	132	(14.0%)	< 0.001^*^	1.016 (0.764–1.342)	0.912
Coronary artery disease	294	(2.8%)	60	(6.3%)	< 0.001^*^	1.175 (0.801–1.703)	0.402
COPD	162	(1.5%)	36	(3.8%)	< 0.001^*^	1.711 (1.080–2.652)	**0.019** ^*^
Cirrhosis	381	(3.6%)	128	(13.5%)	< 0.001^*^	3.861 (2.927–5.072)	**< 0.001** ^*^
Chronic kidney disease	57	(0.5%)	24	(2.5%)	< 0.001^*^	1.938 (0.804–4.584)	0.136
Cancer	197	(1.9%)	35	(3.7%)	< 0.001^*^	1.107 (0.693–1.722)	0.660
RBC transfusion > 20 U^a^	635	(6.0%)	507	(53.7%)	< 0.001^*^	13.285 (11.037–16.025)	**< 0.001** ^*^
Year of injury					0.043		
2002–2005	3438	(32.4%)	301	(31.9%)		1	
2006–2009	3542	(33.4%)	351	(37.1%)		1.152 (0.951–1.397)	0.149
2010–2013	3626	(34.2%)	293	(31.0%)		1.066 (0.872–1.303)	0.534

Multivariate analysis is calculated by logistic regression, () represent 95%confidence interval in odds ration

*PRBC* packed red blood cell

^*^Statistically significant difference (*p* < 0.05)

^a^Taiwan unit, blood product separated from 250 ml of whole blood

## Discussion

The treatment of blunt splenic injuries has been thoroughly discussed over the last two decades, and postsplenectomy immunocompromised patients have remained a concern [8]. Therefore, the NOM of splenic injuries has been advocated [8]. With the considerable development of intensive care units and radiological interventions, the NOM of splenic trauma has become reliable and is now the primary therapeutic modality for splenic trauma. NOM has been successfully used in both pediatric and adult patients [9, 10]. In this study, we assessed the changes in clinical treatments for spleen injuries over the past decade and determined that NOM has increased gradually in tertiary centers and noncenter hospitals.

In tertiary centers, more patients received NOM of blunt splenic trauma than in non- noncenter hospitals. Both institutions had similar clinical outcomes when the severity was analyzed. As shown in Table 2, tertiary centers had a higher percentage of patients with an ISS ≥ 16 than noncenter hospitals during every period of the study. These results are similar to the results of previous studies [11, 12]. The well-established infrastructure, facilities for immediate resuscitation and angiography, 24-h availability of the ICU and confidence of the trauma team might be influencing factors of this result [13].

Table 2 shows that patients with an ISS ≥ 16 tended to be more in recent eras in both groups, which is due to the policy of the NHI program. After 2010, the government of Taiwan encouraged the registration of major trauma for social welfare reasons. Therefore, the number of patients with an ISS ≥ 16 has increased in both tertiary centers and noncenter hospitals. Thus, the severity of trauma might be underestimated in the early years of the study. However, the comparison of severity between the two groups still indicated that the tertiary centers treated more severe patients than noncenter hospitals.

Another controversial point is whether NOM failure increases morbidity or mortality. Previous data showed that NOM failure was not related to mortality [14, 15]. Initially, due to limited experience, NOM might delay the definite treatment and induce massive hemorrhage, which leads to a dismal prognosis in patients with spleen injury [15]. However, recent studies found that conservative treatments were suitable for most people, except for those who were initially hemodynamically unstable and unresponsive to the initial resuscitation [16]. Once the hospital was able to provide immediately necessary rescue procedures, NOM became feasible [3, 17]. Watson et al. reported that mortality was not related to NOM failure. Rebleeding and mortality are not the main complications of NOM, although when these do happen, they elevate the mortality rate to 24% [18]. In our series, the associated injuries did not change over time in either the center or noncenter groups, implying that the severities of patients were similar. Although the in-hospital mortality rates at the tertiary centers were better than those of noncenter hospitals, the OR was 0.94, indicating no difference between the groups.

In the current study, mortality and prognosis were similar in both groups, which deviates from previous results [19]. In our multivariant analysis for the mortality rate of spleen injury, neither NOM nor hospital volume had an impact. This outcome indicated two important concepts. First, spleen injury, although deadly, remains a relatively easily manageable solid organ trauma. Splenectomy is not a difficult surgery and is considered safer for patients in noncenter hospitals that perform less NOM. Second, tertiary centers with more NOM cases had similar outcomes as noncenter hospitals, suggesting that NOM is as safe as surgical intervention for spleen injury. Therefore, regardless of the institution level, if the resources are sufficient, NOM should be considered as the first-line treatment choice.

Doubts still exist regarding the initial criteria for spleen injury, even though the criteria for the NOM of splenic injuries have been established. In a previous study, factors including age, the injury severity, the necessity of blood transfusion, and the presence of associated injury were significantly different between the NOM group and the surgical group [14]. However, we have observed different results. In a geriatric-predominant society such as Taiwan, the elderly comprise more than 40% of the total population. However, age is not a restrictive factor for NOM. In our series, the age difference between the NOM group and the operative group was only 1 year, and no differences in mortality were observed. Other results also showed that the NOM group had significantly lower morbidity rates and shorter hospital stays than the operative group but had equivalent mortality rates to those from other reported series [20].

Prospectively, we noted that Level 1 trauma centers showed better outcomes than did high-volume hospitals, [21] which could explain why the in-hospital mortality rate was almost the same in the center group and non-center group in our series. Our country is an island nation, and some tertiary centers are located in urban areas with a limited number of trauma patients. Thus, some of these tertiary centers have not established a complete trauma system. To improve the trauma management quality, another hospital grading system for trauma should be developed. In addition, there has been discussion about the cost-effectiveness of operative treatments and NOM [22].

Although our study is a national cohort study and presents an obvious increase in NOM of spleen injuries, there are still limitations associated with studies that use the nationwide ICD-9 database. First, we cannot obtain detailed data in terms of the nature of the participants for thorough analysis [23]. For example, although several articles described the utilize of transarterial embolization(TAE) increased the success rate of NOM of a blunt spleen injury. However, the same procedure code of TAE is applied for all anatomical locations. Therefore, we can’t analyze the trend of interventional radiology for splenic injuries precisely in our article. Second, because of the limitations of the NHIRD database, the splenic injury severity cannot be extrapolated to an anatomic injury score or an ISS score; this limitation forced us to use associated injuries, which are recorded in detail, as a replacement assessment score. Despite these limitations, the strengths of our study provide a significant contribution to the analysis of NOM outcomes in modern medical societies.

## Conclusion

In this study, there is a trend toward the use of NOM as a blunt splenic injury treatment, and the outcomes of NOM patients are not inferior to those of surgical patients for all hospital levels. Therefore, NOM should be considered the primary treatment choice once the hospital is confident of their definite treatment capacities. In addition, tertiary centers performed more NOM than noncenter hospitals and better met the international consensus.

## Additional file

Additional file 1: **Table S1.** International Classification of Diseases, Ninth Revision, Clinical Modification (ICD-9-CM) codes used for diagnosis, associated injury, underlying disease and complications in current study. (DOCX 40 kb)
