# Supplementary material for: Hospital level variations in the trends and outcomes of the nonoperative management of splenic injuries – a nationwide cohort study
Source: Scand J Trauma Resusc Emerg Med. 2019 Jan 11;27:4. doi: 10.1186/s13049-018-0578-y (PMC6329069; doi:10.1186/s13049-018-0578-y)
Supplement: Supplementary file 1 — Table S1. International Classification of Diseases, Ninth Revision, Clinical Modification (ICD-9-CM) codes used for diagnosis, associated injury, underlying disease and complications in current study. (DOCX 40 kb) [file 13049_2018_578_MOESM1_ESM.docx]

**Supplementary Table 1.** *International Classification of Diseases, Ninth Revision, Clinical Modification* (ICD-9-CM) codes used for diagnosis, associated injury, underlying disease and complications in current study

| **Diagnosis** | **ICD-9-CM code** |
| --- | --- |
| **All spleen injury** | 865.xx |
| **Penetration spleen injury** | 865.1x |
| **Associated injury** |  |
| Traumatic brain injury | 850.3x-850.5x, 850.9x, 851.xx-854.xx, 800.xx-804.xx |
| Cardiopulmonary injury | 861.0x-861.1x, 861.2x-861.3x |
| Hemothorax | 860.2x-860.5x |
| Gastrointestinal injury | 863.xx |
| Kidney injury | 866.xx |
| Liver injury | 864.xx |
| Pelvic fracture | 808.xx |
| Femoral fracture | 820.xx-821.xx |
| Spine fracture | 805.xx-806.xx |
| **Underlying disease** |  |
| Diabetes mellitus | 250.xx |
| Hypertension | 405.xx-405.xx |
| Coronary artery disease | 410.xx-414.xx |
| COPD | 491.xx, 492.xx, 496.xx |
| Cirrhosis | 571.2x, 571.5x, 571.6x |
| Chronic kidney disease | 585.xx |
| Cancer | 140.xx-208.xx in Catastrophic illness database |
| **Complications** |  |
| Pneumonia | 480.xx-486.xx |
| Wound infection | 998.5x, 958.3x |
| Other infection | 038.xx, 440.24, 590.10, 599.0x, 682.6x, 707.0, 728,86, 730.27, 785.4, 790.7, 996.62, 997.62 |
| Stroke | 433.xx-437.xx |
| Gastrointestinal bleeding | 530.21, 530.7, 530.82, 531.xx-535.xx, 537.83-84, 578.xx |

†Patient with malignant disease is one of indications to apply catastrophic illness certificate and will be registered in catastrophic illness database.
